# Topological Anisotropy of Stone-Wales Waves in Graphenic Fragments

**DOI:** 10.3390/ijms12117934

**Published:** 2011-11-15

**Authors:** Ottorino Ori, Franco Cataldo, Mihai V. Putz

**Affiliations:** 1 Actinium Chemical Research, Via Casilina 1626/A, 00133 Rome, Italy; E-Mail: franco.cataldo@fastwebnet.it; 2 Laboratory of Computational and Structural Physical Chemistry, Chemistry Department, West University of Timişoara, Pestalozzi Street No.16, Timişoara, RO-300115, Romania; 3 Research Center for Einstein Physics, Institute of Theoretical Physics, Free University Berlin, Arnimallee 14, 14195 Berlin, Germany

**Keywords:** topological modeling, Wiener index, Stone-Wales wave, carbon nanostructure

## Abstract

Stone-Wales operators interchange four adjacent hexagons with two pentagon-heptagon 5|7 pairs that, graphically, may be iteratively propagated in the graphene layer, originating a new interesting structural defect called here Stone-Wales wave. By minimization, the Wiener index topological invariant evidences a marked anisotropy of the Stone-Wales defects that, topologically, are in fact preferably generated and propagated along the *diagonal* of the graphenic fragments, including carbon nanotubes and graphene nanoribbons. This peculiar edge-effect is shown in this paper having a predominant topological origin, leaving to future experimental investigations the task of verifying the occurrence in nature of wave-like defects similar to the ones proposed here. Graph-theoretical tools used in this paper for the generation and the propagation of the Stone-Wales defects waves are applicable to investigate isomeric modifications of chemical structures with various dimensionality like fullerenes, nanotubes, graphenic layers, schwarzites, zeolites.

## 1. Introduction

**Figure 1:**
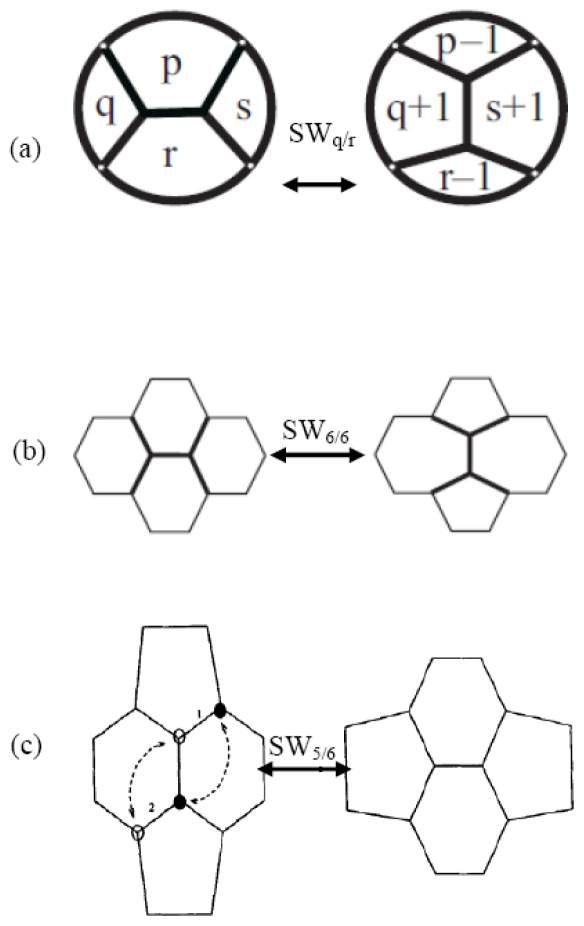
(**a**) Local transformation SW_q/r_ changes a group of four proximal faces with *p, q, r*, *s* atoms in four new rings with *p*−1*, q+*1*, r*−1, *s+*1 atoms; (**b**) On the graphene layer (*p=q=r*=*s=6*) SW_6/6_ reversibly flips four hexagons in a 5|7 double pair; (**c**) SW_5/6_ reversible flip on the fullerene surface.

On hexagonal systems like graphene layers, graphene nanoribbons (GNR’s) and carbon nanotubes (CNT’s), the isolated pentagon–heptagon *single* pair (also called 5|7 pair, 5|7 defect, 5|7 dislocation or the *pearshaped* polygon [1]) and pentagon–heptagon *double* pair 5/7/7/5 arising from the celebrated Stone-Wales transformation (SW transformation or SW rotation [2]) are important structural defects largely influencing chemical, mechanical, and electronic properties [3]. Figure 1 represents the general Stone-Wales transformation SW_q/r_ (Figure 1a), associated to the most studied variants, the SW_6/6_ in graphene (Figure 1b) often called Stone-Thrower-Wales rotation and the SW_5/6_ in fullerenes (Figure 1c) the so-called pyracylene rearrangement.

Scope of this theoretical article is to illustrate the topological properties of SW rotations in hexagonal systems investigating, in particular, the family of isomeric SW transformations able to *generate and propagate* 5|7 defects in graphenic fragments, graphene nanoribbons and carbon nanotubes. We present initially (see next paragraph) an original graphic tool able to modify the hexagonal patterns of carbon atoms under the action of subsequent SW bond rotations generating 5 and 7-membered carbon rings. Our tool operates in the dual space and, more generally, it creates various kinds of defective layers with no limitations on the composition of the modified rings that may have any number of members *m* = 3, 4, 5, 6, … The topological simulations confirm moreover that SW double pairs 5/7/7/5 possess a peculiar anisotropy, matching, from a pure *topological* point of view similar *ab-initio* results on sp^2^-carbon systems recently appeared in literature (see [4] and related).

Another interesting topological effect is also introduced, consisting in the diffusion of a 5|7 pair in the hexagonal network as a consequence of iterated SW rotations; this topology-based mechanism *that produces a linear rearrangement of the hexagonal mesh* is called here the *SW wave*. Whereas mechanically exfoliated monolayer graphene is structurally (almost) perfect in atomic scale [3], graphene layers produced by chemical vapour deposition (CVD) techniques present a parade of defect structures, which are due to the growth on substrates with surface defects and/or other irregularities. New stable carbon allotropes have been therefore proposed [5] by considering the presence of periodical arrangements of defective building blocks such as Stone-Wales defects, inverse Stone-Wales defects, vacancy defects, and other structural modifications of the pristine hexagonal plane. The first experimental observation of a particular type of linear topological defects is reported in [6] where extended chains of octagonal and pentagonal sp^2^-hybridized carbon rings, detected by scanning tunneling microscopy (STM) images, function as a quasi-one-dimensional metallic wire and may be the building blocks for new all-carbon electronic devices. This important experimental finding enforces meanwhile the theoretical role of the SW waves, that are in principle structurally simpler than the pentagons-octagons chain reported in [6], as a possible *hexagonal inter-grain spacing* (see the visualizations given in Section 2) between graphenic fragments. Molecular mechanics simulations show that in graphene the presence of cylindrical curvature energetically facilitates such a split of the 5/7/7/5 SW dislocation dipole [4], assigning to this class wave-like atomic-scale rearrangements a fundamental role in nanoengineering of graphenic lattices. One has however to notice that other transmission electron microscopy (TEM) detailed measurements point out [7,8] that the migration and the separation of the pentagon-heptagon pairs does not happen on planar graphene membranes where the 5–7 defects relax back reconstructing the original graphene lattice. These experiments indicate that extended *dislocation dipole*, favored by the presence of structural strain, preferably appear in curved graphitic structures or systems like CNT or fullerene molecules. In epitaxial graphene grown at high temperatures on mechanically-polished SiC(0001), a characteristic 6-fold “flower” defect results from STM measures [9,10]. We note that the observed rotational grain boundaries is conveniently describable as *radial* type of the *SW wave* suggesting that the wave-like theoretical mechanism presented here, may have a general applicability.

The SW rotations applied in the present studies derive from the general (Figure 1a) Stone-Wales local and isomeric transformation SW_p/r_ varying the internal connectivity of four generic carbon rings made of *p, q, r*, *s* atoms to produce four new adjacent rings with *p*−*1,q+1,r*−*1*,*s+1* atoms without changing the network of the surrounding lattice. SW_p/r_ reversibly rotates the bond shared by the two rings *p* and *r*, preserving both, the total number of carbon atoms

v=p+q+r+s-8

and the total number of carbon-carbon bonds

e=v+3

On the graphene ideal surface, made only of hexagonal faces, the SW_6/6_ rotation transforms four hexagons in two 5|7 adjacent pairs (Figure 1b) symbolized in literature [4,11] as 5/7/7/5 defect and also quoted as the *SW defect* or the *dislocation dipole*. We remember here that the SW rotations play an important role in connecting the isomers of a given C*_n_* fullerene with different symmetries. In the crucial case of the C_60_ fullerene, its 1812 isomers are grouped by the *pyracylene* rearrangements SW_5/6_ (Figure 1c) in 13 inequivalent sets (the larger one consisting of 1709 cages) connected to the buckminsterfullerene (C_60_-I_h_) through one or more SW transformations [12], leaving 31 isomers unconnected to any of these sets. This limitation has been overcome by the introduction of non-local generalized Stone-Wales transformations [13] to generate the whole C_60_ isomeric space starting from just one C_60_ isomer.

Theoretical investigations based on plane-wave density-functional methods [12] set to no less than 6.30 eV the uphill energy barrier dividing the buckminsterfullerene from the SW connected isomer with C_2v_ symmetry; this barrier reaches 9 eV for hexagonal systems like nanotubes or large graphene portions. Using the extended Hückel method, enlarging the relaxation region around the SW defect, it can be found that the formation energy of a SW defect considerably decreases to 6.02 eV for a flat graphene fragment case. This result has been verified by using *ab initio* pseudopotential [14]. This result seems to preclude the formation of any SW 5/7/7/5 defect in nature, but as it has been reported [15,16] that this barrier drops rapidly, reducing to 2.29 eV the creation barrier of SW rotations due to the catalyzing action of interstitials defects or ad-atoms present in the hexagonal networks. Pentagon–heptagon pairs have been predicted to be stable defects also in important theoretical articles [17,18] showing that energetic particles, as electrons and ions, generate 5|7 pairs in graphite layers or CNT’s as a result of knock-on atom displacements. On the experimental side, accurate high-resolution TEM studies made on single-walled carbon nanotubes [19] or electron-irradiated pristine graphene [20] document *in situ* formation of SW dislocation dipoles. TEM measures also evidence [21] stable grain boundaries with alternating sequence of pentagons and heptagons that show the relevance of wave-like defects during graphene edge reconstruction.

Extended theoretical investigations [22] by means of first-principles density-functional computations, demonstrate that, on graphene layers, the dislocation dipole 5/7/7/5 defects become particularly stable - in comparison to other possible local defective structures as haeckelite units with three pentagons and three heptagons—when the two 5|7 pairs are separated by lattice vacancies in the number of ten or over. Moreover, recent literature (see the excellent review [3]) on GNR’s constructed from haeckelites considers systems with SW defects as new hypothetical nano-architectures with fascinating applications in electronics. Isolated 5|7 pairs could also appear at grain boundary in graphene fragments, changing their edge termination and electronic properties, forming *hybrid* GNR’s. These hybrids exhibit half metallicity in the absence of an electric field, and could be used to transport spin-polarized electrons; which could be a step forward in new spintronic devices.

Considering the above experimental and theoretical evidences of the structural stability of hexagonal systems with 5|7 defects, this theoretical note aims to investigate the topological, wave–like mechanisms leading the diffusion (or annihilation) of pentagon–heptagon pairs.

**Figure 2:**
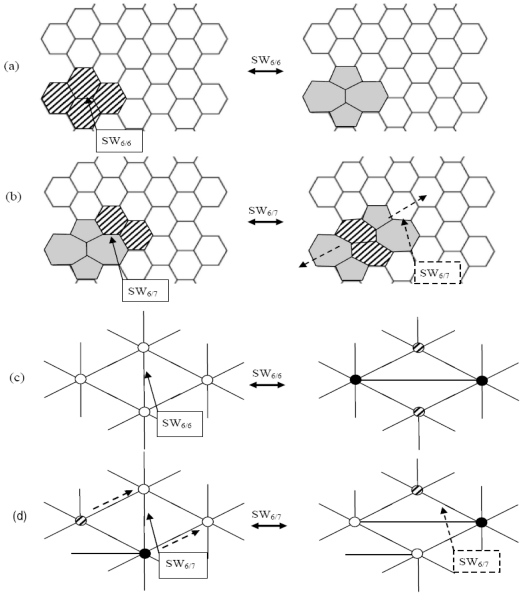
(**a**) SW_6/6_ originates two 5|7 pairs (in gray); (**b**) SW_6/7_ splits the pairs by swapping one of them with two nearby hexagons (shaded). Dotted SW_6/7_ pushes the SW wave in the dashed direction; (**c-d**) Mechanisms (a,b) in the graphene dual plane. Hexagons, pentagons, heptagons are represented by white, shaded, black circles respectively.

Figure 2 shows the fundamental topological operations for the generation and the propagation of SW waves in the graphene lattice. The first rotation SW_6/6_ (Figure 2a) of the chemical bond (arrowed) shared by the two hexagons creates the two 5|7 pairs (the SW defect 5/7/7/5). The second operator SW_6/7_ turns the bond between the heptagon and the nearby shaded hexagon and inserts the 6|6 couple of shaded hexagons between the two original 5|7 pairs (this topological defect is also referenced in [4] as 5/7/6/6/7/5), leading to the overall structural effect of initiating the propagation of the SW wave along the dotted direction (Figure 2b). Iterated transformations SW_6/7_ will successively drift the 5|7 pairs in the lattice (along the dotted directions in (Figure 2b), producing the topological SW wave (SWW).

SWW mechanism provides theoretical support to recent studies on graphenic structures. Some authors [1] emphasize the importance of 5|7 dislocations monopole at the grain boundaries of polycrystalline graphene, stating that these defects cannot be annealed by any local reorganization of the lattice. SW waves allow 5|7 dislocations also to anneal by just involving surrounding 6|6 pairs and moving *backward*, being all transformations in Figure 2 completely *reversible*.

Theoretically, SW defects and isolated 5|7 pairs have been extensively investigated in [11] where *ab-initio* simulations of the electronic properties are reported; authors conclude that a single heptagon–pentagon dislocation is a stable defect whereas the Stone-Wales adjacent pairs are dynamically unstable. These two conformations may easily find a unified description considering that these lattice defects correspond to different propagation steps of the same SW wave.

Considering the very rich variety and complexity of all possible paths that SW waves may describe on the graphenic surface, involving a variable numbers of 5|7 pairs, this article just focuses on the topological properties exhibited by the linear propagation of the basic SW defect, the 5|7 double pair (Figure 1b). This choice limits the SW_p/r_ rotations to just to the operators SW_6/6_ and SW_6/7_. In spite of the apparent simplicity of our model, *SW waves present* an evident and *marked topological anisotropy* immediately signaled by the Wiener index [23] *W*(*N*) of the graphenic system under study (graphene fragments, CNT’s and GNR’s).

It is really important to note that, more and more, various anisotropic effects are evidenced in literature [3,4,24–27] by applying first-principle techniques to the determination of the energy-stress behaviors of different configurations of SW defects on graphene nanotubes and nanoribbons. Similar effects appear in the theoretical distribution of magnetic dipoles in defective carbon metallic nanotubes [28]. A comparison between our findings and literature results is also provided.

## 2. Generation and Propagation of Stone-Wales Rearrangements

**Figure 3:**
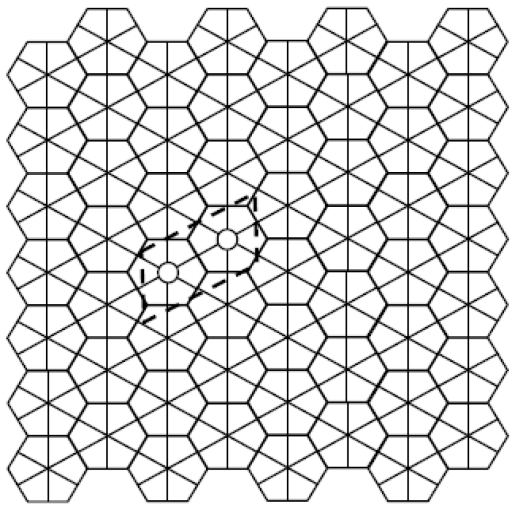
Dual representation of the graphene lattice obtained by replacing each hexagonal face by the central 6-connected graph node. Graphene plane is then equivalently tiled by hexagons (direct space) or by starred nodes (dual space). The x-periodic (y-periodic) direct graphene nanoribbon has the armchair (zig-zag) orientation. The framed unit cell has been used to build this 4 × 7 graphenic fragment.

Before modeling the SW wave propagation, it is worth introducing the graphic tool used to generate this kind of defects on the hexagonal structures. The effectiveness of such an algorithm derives from the choice to operate in the *dual topological representation* of the graphenic layers as shown in Figure 3. Also the topological modeling will be conducted in the dual space.

The generation of the SW rotations is greatly facilitated by considering the dual representation of the graphene layer by assigning to each hexagonal face the corresponding 6-connected (starred) vertex. Figure 3 visually overlaps both direct and dual graphene representations showing their topological equivalency: each pair of adjacent faces in the direct lattice corresponds in fact to a pair of bonded nodes in the dual graph and vice versa. The graphene fragment is taken in its *armchair* orientation along *x* and Figure 3 evidences a 4 × 7 dual lattice and its unit cell. In the dual lattice the generic SW_p/r_ *simply rotates* the internal edge between the *p*- and the *r*-connected nodes, making the study of the SW rearrangement very simple and suitable for automatic procedures.

On the graphene dual layer the SW_6/6_ rotation (Figure 2c) then changes four 6-connected nodes (white circles) into two 5-connected (shaded circles) and two 7-connected (black circles) vertices, matching the standard transformation in the direct lattice of four hexagons in two pentagons and two heptagons (Figure 2a). Moreover 5|7 pairs may also *migrate* in the graphene lattice, pushed by consecutive Stone-Wales transformations of SW_6/7_ type that rotate, in the dual space, the *vertical* edge between the 6-, and the 7-connected vertices, driving the *diagonal* diffusion of a 5|7 pair in the graphene lattice. Figure 2d gives more details about the swapping mechanism between the 5|7 and the 6|6 couples. The repeated action of the SW_6/7_ operator originates the topological SW wave in both lattice representations.

**Figure 4:**
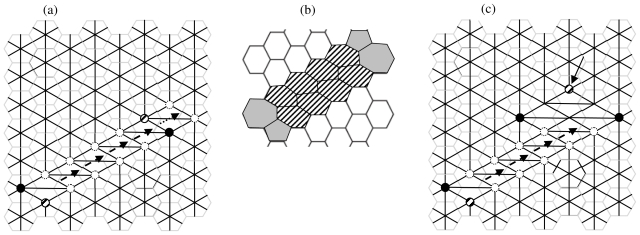
(**a**) Diagonal SW wave (dislocation dipole) in the dual graphene layer after the generation and four propagation steps (size *η* = 4); at each step (dashed arrows) SW_6/7_ swaps the pair made by one pentagon (dashed circle) and one heptagon (black circle) with two connected hexagons (dotted circles); dotted arrow indicates the next available translation of the 5|7 pair; (**b**) The topological modification (a) originates, in the direct lattice, a hexagonal inter-grain spacing (dashed rings); (**c**) After a few more SW rotations, an isolated pentagon (arrowed), forming a small nanocone, is generated.

Figure 4a represents the diagonal diffusion of the SW wave (dislocation dipole) after four SW_6/7_ rearrangements, evidencing with the dashed arrows the increasing distance between the two 5|7 pairs of the original SW_6/6_ dislocation. At each step, the pentagon (shaded circle) and the heptagon (black circle) interchange their locations with those of two hexagons (white circles) producing the *diagonal SW wave*, a large dislocation dipole that modifies the landscape of direct and dual lattices (Figures 4a and 4b). Being *η* the size of the dislocations (e.g., *η* equals the number of 6|6 pairs included between the two 5|7 pairs) both examples in Figure 4 have size *η* = 4, assuming size *η* = 0 for the basic SW_6/6_ rotation of (Figures 1b). Equivalently, *η* equals the number of SW_6/7_ rearrangements used to generate the dislocations in both spaces. A SW wave produces (Figure 4b) a characteristic *hexagonal inter-grain spacing*, isomeric to the pristine graphene layer that represents therefore a good theoretical model for the boundary between graphenic fragments.

**Figure 5:**
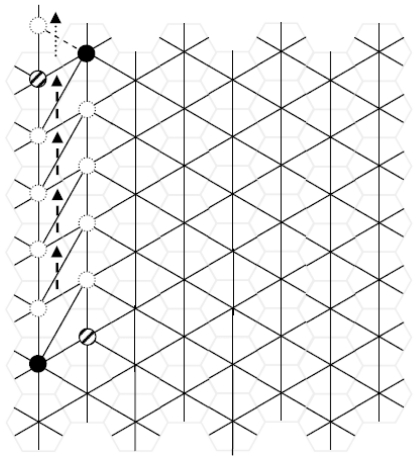
SW vertical wave in the dual graphene layer after four propagation steps (dashed arrows); SW_6/7_ swaps the pentagon (dashed circle) heptagon (black circle) pair with two hexagons (dotted circles); dotted arrow indicates the next possible translation of the 5|7 pair, induced by a SW_6/7_ rotation of the hexagon-heptagon diagonal dashed bond. The SW wave generates anti-diagonal hexagons-hexagons bonds with respect to the unrotated one.

The dual space represents the natural arena for studying all sorts of SW flips, avoiding the graphical difficulties that one usually encounters in redistributing the carbon atoms and bonds in the direct lattice. One easily generates the *vertical* SW wave by applying in fact our graphical algorithm to the *diagonal* edges of the graphene dual lattice (Figure 5). SW transformations produce also very complex rearrangements of the graphenic layer including isolated pentagonal nanocones, as the very little one on top of the diagonal SW wave in Figure 4c, creating fullerenic-like regions in the graphenic plane [29]. The proposed dual space graphical algorithm appears therefore capable to handle complex combinations of general Stone-Wales rotations SW_p/r_ to create novel classes of isomeric rearrangements, with rings made of various numbers of atoms, of fullerene (dimensionality *D* = 0), nanotubes (*D* = 1), graphenic structures (*D* = 2) or crystals (*D* = 3) as schwarzites or zeolites.

The some-how arbitrary definition of *diagonal* or *vertical* direction assigned to the SW waves on closed surfaces of graphenic fragments, nanoribbons, nanotubes considers the *armchair* graphene orientation selected in Figure 3. Topologically, the extension of the region interested by the dislocation dipole may be arbitrarily enlarged by applying more and more SW_6/7_ rotations.

Energetically, the situation is more articulated; as explained in the next Section, the lattice shows in fact anisotropic reactions to the propagation of the SW waves along different directions when a closed graphene fragment is considered.

In summary, on the armchair-oriented graphene (Figure 3), the simplest propagation mechanisms available for the 5|7 pairs are:

- *Diagonal* SW wave, Figure 4: SW_6/7_ rotates the *vertical* bond of the graphene dual lattice between the 6-connected node and the 7-connected node of the diffusing 5|7 pair, causing the *diagonal* drift of the pair and the creation of a new horizontal hexagon-hexagon bond (Figure 2d gives some more details);
- *Vertical* SW wave, Figure 5: SW_6/7_ rotates the *diagonal* bond of the graphene dual lattice between the 6-connected node and the 7-connected node of the diffusing 5|7 pair, with the overall effect to *vertically* shift the pair, generating a new *anti-diagonal* hexagon-hexagon bond.

Above diffusion processes apply to an isolated 5|7 dislocation monopole as well to the 5|7 double pair arising from a SW_6/6_ rearrangement. In the following we mainly study this latter case, focusing on the mechanisms leading to the creation of diagonal or vertical *extended dislocation dipoles* in the graphene lattice.

It is worth noting that similar topological tools are used in other disciplines like in Biology where wave-like diffusion mechanisms model cells proliferation processes [30].

In concluding this Section we observe that from the pure topological point of view one may consider each lattice configuration illustrated in this work as the result of an *instantaneous* transformation caused by a *single, non-local* SW rotation. This new class of transformations represents a further generalization, potentially infinite, of the non-local rearrangements early proposed [13] to generate the entire isomeric space of a given C*_n_* fullerene starting for a limited number of inequivalent cages.

## 3. Theoretical Basis of the Topological Model

The nature of the SW waves is investigated here by means of graph-theoretical methods only, postponing the correlations to the energy of the system to future specific investigations on the subject. According to this approximated model, we assign to the Wiener index *W*(*N*) topological invariant [23] the role of *topological potential* of the system, subject to a minimum principle. This approximated model assumes that similar carbon systems have the tendency to arrange their structures minimizing the invariant *W* seen as the inteartomic, long-range potential among all pairs of carbon atoms. Heuristically, this approach is confirmed from the fact that, for example, among 1812 non-isomorphic C_60_ fullerene isomers, just the *physically stable* isomer with icosahedral symmetry C_60_-I*_h_* and isolated pentagons corresponds to the *isomer with the minimum W value W* = 8340 and the highest *topological compactness*. This concept is naturally extended to the isomers of any other carbon chemical systems. Our simulations aim therefore for the *most-compact structures seen as very good candidates for chemically stable systems*. [29,31] present recent successful applications of this method to graphenic layers and C_66_ fullerene. Computationally, our assumption implies the topological minimum principle on *W: chemically stable structures have to be searched among the configurations minimizing the W index*.

Current selection of the topological potential, privileges transformations of the graphene layer, increasing the system compactness. The same method has been recently used in simulating the growing steps of fullerene-like nanostructures on the graphene dual plane [29] or the stability of the C_66_ fullerene [31] with a good match with the experimental results.

For a chemical graph with *N* vertices, *W* comes from the half-sum of the chemical minimum distances *d_ij_* between all pairs of vertices *V_i_, V_j_* in the lattice:

(1) $$W(N)=\frac{1}{2}\sum_{ij}d_{ij}$$

On large structures, this distance-based invariant shows a remarkable polynomial behavior. For infinite one-dimensional graphs [32] (polymers) it grows as *W*(*N*) ≈ *N*^3^, being that a particular case of the polynomial-like general formula *W*(*N*) ≈ *N*^s^ (with *s =*2 + 1*/D*) recently conjectured for large *D*-dimensional lattices [29]. In case of *D =* 2 structures, the general closed form for the Wiener index is:

(2) $$W(N)=b_{5}N^{5/2}+b_{4}N^{2}+\ldots +b_{1}N^{1/2}+b_{0}$$

For the dual graphene lattice in Figure 3 the following elegant relation holds [33]:

(3) $$W(N)=\frac{1}{\sqrt{2}}\frac{7N^{5/2}-2N^{3/2}}{24}$$

where *N* represents the number of vertices of the dual lattice e.g., the number of hexagonal faces in the graphenic layer. Periodic boundary conditions are imposed on the dual graphenic lattices along the current study. The influences of the topological potential *W* on the propagation of the diagonal and vertical SW waves in the graphene dual lattice are discussed in the next paragraph.

## 4. Results and Discussions

Initially, the topological propagation of the SW waves has been simulated on the *G_10_* dual graphene lattice consisting of *10 ×10* unit cells with *N = 200* starred vertices and periodic boundary conditions. Equation (3) attributes to that ideal closed lattice *G_10_* the reference value of the topological potential *W_G10_* *= 116,500*.

Let’s now generate and propagate the diagonal SW wave (in Figures 4a and 4b).

The first diagonal SW_6/6_ flip produces the 5|7 double pair (Figures 2a and 2c) and decreases the lattice potential to *W = 116,015*, easily derivable from the direct computation of the graph chemical distances according to the definition (1). In our approximated model this negative 0.42% variation of the Wiener index represents the *topological gain* induced by the creation of a *diagonally oriented* SW defect.

**Figure 6:**
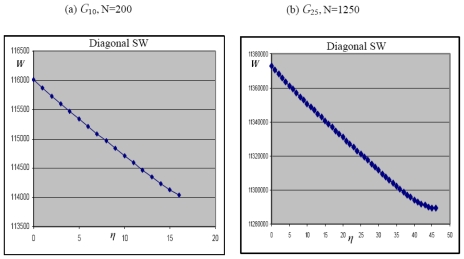

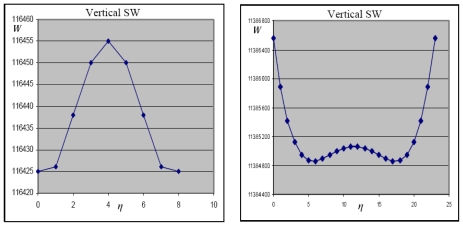
(**a**) Wiener index *W* for diagonal and vertical SW waves for the *N = 200* dual closed graphene graph *G*_10_ as a functions of the wave propagation steps *η*. Diagonal dislocation dipoles (top) freely flow in the lattice, whereas the vertical ones (bottom) are stopped; (**b**) On the *N* = 1250 lattice *G*_25_, vertical SW waves (bottom) present a limited penetration (*η* = 6), being the diagonal penetration of the defects still favored (top). The ratio *W_G_*_25_*/W_G_*_10_ ≈ 97.77 between the *W* values for the two ideal lattices *W_G_*_25_ *=* 11390625 and *W_G_*_10_ *=* 116500 follows the ratio of the *N_5/2_* leading terms in Equation (3).

The subsequent SW_6/7_ rotation (Figures 2b and 2d) translates one of the 5|7 pair with a further decrease of the topological potential being *W = 115 870* for the step *η* = 1 on *G_10_*. This behavior is confirmed at each propagation steps of the diagonal SW wave, augmenting the topological stability of the system. The reduction of the *topological potential W* follows an almost linear trend, see the top curve of (Figure 6a) where the number of propagation steps *η* is reported, the *η* = 0 case corresponding to the creation of the diagonal dislocation SW dipole with topological potential *W = 116,015*. The result evidences the tendency of the *10 × 10* graphene fragment to allow the unlimited topological diffusion the 5|7 pair *along the diagonal* direction with the creation of extended diagonal dislocation dipoles (Figures 4a and 4b). This characteristic of the diagonal SW wave has a *pure topological root* strongly correlated to the connectivity properties of the pentagon-heptagon pairs embedded in the hexagonal mesh and to the edge effect induced by the fragment boundary.

A different situation is encountered by simulating the vertical SW wave given in Figure 5 that moves in the graphene fragment parallel to the ribbons of hexagons, orthogonally to the armchair edges.

It is worth noticing that both the heptagon-pentagon and hexagons-hexagons bonds generated by the vertical SW in the dual lattice form a π/3 angle with the armchair edge, see Figure 5. After the first SW_6/6_ vertical rotation, the two new 5|7 pairs give *W = 116,425* for *η* = 0 with a little topological gain of just −0.06% compared to *W_G10_* *= 116,500* of the pristine lattice, smaller than the one (−0.42%) detected in the diagonal case. The successive SW_6/7_ flip starts the vertical propagation (*η* = 1) of one of the 5|7 defect, slight increasing the topological potential *W = 116,426*. This growth of the topological potential opposes to the vertical diffusion of the wave, being this barrier effect confirmed at the successive steps *η* = 2 and *η* = 4 by the increasing values *W = 116,438* and *W = 116,455*. According to our simulations therefore the topological potential *W* obstacles (Figure 6a bottom) the diffusion of the vertical SW wave in the closed *10 × 10* graphene fragment *G*_10_.

The influence of the size of the system on the reported topological anisotropy has been investigated by considering the closed dual graphene lattice *G*_25_ with *N = 1250* starred nodes and starting value *W_G25_* *= 11,390,625*. After the first SW diagonal rotation (*η* = 0) the potential passes to *W_η_*_=0_ *= 11,373,153* with a gain in the topological stability of about −0.15%. One may also observe the vertical propagation on the G_25_ lattice suffuses that of G_10_ one in accordance with the idea that for infinite extended system it will resemble the potential well, within which the diagonal movement takes place. The *N_5/2_* leading terms in Equation (3) give for the ideal lattices with *N* = 1250 and *N* = 200 an approximated ratio of 97.66 that matches quite well the corresponding fraction *W_G25_/W_G10_* ≈ 97.77 showing the fast convergence of the Wiener index polynomial (3).

It is moreover very interesting to note that a similar ratio 98.03 characterizes also the previously reported *W* values of the defective *G*_10_ an *G*_25_ layers with one SW defect (*η* = 0) suggesting that an infinite set of exact polynomial functions *W(N, η)* may be found as generalization of the Formula (3) to describe—still with the *N_5/2_* dependence—the topological potential *W* in presence of diagonal (or vertical) dislocation dipoles with variable size *η*; this topological property will be the subject of future investigations.

Our simulations on the *G*_25_ lattice confirm that the diagonal propagation on large distances of the 5|7 pair is still favored (Figure 6b top) whereas just a limited penetration (*η* = 5) of the vertical SW dislocation dipole is allowed (Figure 6b bottom). This behavior differs from the sharp potential barrier encountered by the vertical SW wave in the smaller *G*_10_ layer (Figure 6a bottom) and one may consider the limited vertical propagation in *G*_25_ as the tendency of the system to recover, for large *N*, the equivalency between the two plane directions that, for the infinite graphenic sheet, are totally indistinguishable.

The anisotropy of the SW waves constitutes an important effect induced by the topological potential *W* on the closed graphenic systems studied here and complements the quantum mechanical origin, as suggested by recent researches on carbon nano-ribbons (GNR’s) [24,25].

The main achievements of the present results are summarized as follows:

- The tendency of the 5|7 defects [19,22] to cover large graphene regions find here a specific topological mechanism, the SW wave. It produces (via the initial SW_6/6_ flip) two 5|7 pairs and then separates them via consecutive SW_6/7_ rotations, creating an extended *dislocation dipole*; the reversed isomeric operation may also take place to annihilate distant 5|7 pairs;
- In the graphene layer SW_6/7_ flips are also able to transport isolated 5|7 dislocation monopoles, by exchanging the heptagon and pentagon positions with those of two nearby hexagons; this drifting mechanism may also annihilate or modify the 5|7 pair in colliding with other structural defects (grain boundaries, other 5|7 pairs, generic *q*|*r* pairs, *etc.*). This result integrates previous studies [1] providing the invoked local annealing mechanism.

The preference for diagonal SW waves perfectly matches results in literature [22] based on density-functional theoretical methods describing large (*η* ≥ 5) *diagonal* dislocations dipoles as particularly stable lattice configurations. These results provide a sound theoretical base to our approximated topological model. Further studies [4] based on molecular mechanics methods confirm the existence of anisotropic diffusion mechanisms in hexagonal systems in presence of *multiple SW defects*. In [4] is demonstrated that the *diagonal* distribution of multiple 5/7/7/5 dipoles is energetically favorable in graphenic lattices differently curved, including nanotubes. Moreover, the presence of the cylindrical curvature, associated to small tensile strain, causes [4] the diffusion of the defects with the insertion of a certain number η of 6|6 hexagon-hexagon pairs between the two 5|7 pairs of the initial 5/7/7/5 SW dipole, showing that the diagonal propagation of the SW wave is energetically favored in (curved) hexagonal systems; these results represent a first theoretical validation our topological model and future investigations will be expressly devoted to correlate the topological potential represented in Figure 6 with the energy of planar and curved graphenic layers. We observe that the instrumental role, in *curved* graphitic structures, of the induced tensile strain in allowing the creation and the diffusion of dislocation dipoles has been recently confirmed by extended experimental and theoretical studies [7,8], stating that SW defects form in graphene with a lower probability than in CNT’s.

A further confirmation of the existence of a preferred direction for the diffusion of the SW pairs comes from the distinct pentagon-pentagon bond energy predicted in [27] for the two different orientations of the SW defect in (5,5) single-walled carbon nanotubes; these results increase the chemical relevance of to the anisotropic propagation mechanisms of the Stone-Wales waves proposed here as an edge effect characterizing hexagonal nanosystems, as GNR’s, CNT’s and large graphene fragments like the above *G*_25_ lattice or the 6344 atoms square graphite sheets previously modeled in literature using molecular mechanics tools [4]. In ref. [28] the characteristic spatial patterns of electric current flow are studied in metallic arm-chair CNT’s depending on the orientation of the SW defect. The presence of rotating loop currents at nanometer scale is originated by quantum interference of conducting and quasi-bound states of electrons in the region of the dislocation dipole, and generates typical patterns of induced magnetic dipoles suitable for experimental detection. The distribution of the loop currents effectively distinguishes the symmetry of the SW defects suggesting that this anisotropic magnetic effect may occur in a general nanostructure, finding potentially application in novel electronic and magnetic nanodevices. Electronic and chemical properties of 5|7 or 5/7/7/5 topological defects are different from the ones exhibited by structural defects (e.g., the presence of single non-hexagonal rings surrounded by hexagonal rings) and, according to review [3], “their reactivity and detection needs to be investigated theoretically and experimentally”. Article [21] gives an interesting evidence of a possible topological SWW mechanism, showing the linear defect that appears during the TEM edge reconstructions of a graphene sheet under the effect of a 80 kV transmission electron microscopy. The stable edge configuration, made of an alternating sequence of pentagons and heptagons, swaps with the pristine zigzag edge, the energy input from the beam proving the required activation energy.

These studies reflect the relevance of the anisotropic behavior of SW defects and of the topological SW waves mechanism both introduced in this article. Further investigations, both theoretical and experimental, are required to fully understand the capability of the suggested mechanisms in producing stable edge reconstructions in graphenic systems.

Finally, we notice that the present topological model is applicable to a wide class of chemical structures including systems with vacant atoms or other kinds of structural defects or to describe the evolution, driven by the topological potential *W*, of C*_n_* nanoflakes in which 5|7 pairs are stable defects according to energy-minimization technique [34].

## 5. Conclusions

The proposed topological potential *W* effectively simulates the diffusion of 5|7 pairs in graphenic structures, favoring the new diagonal wave-like mechanism (SW wave) whose importance has to be assessed by future experimental and first-principle studies. The reported anisotropy represents a peculiar property of the SW defects population of graphenic limited portions, nanotubes, *etc*.

The dual representation of the systems makes the generation and the characterization of SW waves remarkably easy, allowing the fast iteration of arbitrary sequences of generic SW*_q_*_|_*_r_* rotations to produce an endless, at the moment largely unknown, sequence of new isomeric configurations in chemical structures with various dimensionality like fullerenes, nanotubes, graphenic layers, schwarzites, zeolites.

An open question remains regarding the infinite iteration of steps of SW wave propagation that should stabilize the net to a given energetic value, with observable character; this may be treated through combining the present topological approach with the path-integral or propagator information [35] contained therein and to provide the allied quantum information (e.g., the canonical partition function) that allows for evaluation of all thermodynamic functions, phase-transition included. Nevertheless, this will be the subject of forthcoming communications.
